# *Trichomonas vaginalis:* Diagnosis and Clinical Characteristics in Pregnancy

**DOI:** 10.1155/S1064744994000141

**Published:** 1994

**Authors:** R. Phillip Heine, James A. McGregor, Elisa Patterson, Deborah Draper, Janice French, Ward Jones

**Affiliations:** 1 Department of Obstetrics and Gynecology University of Colorado Health Sciences Center Denver CO USA; 2 University of Colorado Health Sciences Center 4200 East Ninth Avenue Box B198 Denver CO 80262 USA

## Abstract

*Objective:* The objectives of this study were to 1) determine the prevalance and characterize the symptomatology of *Trichomonas vaginalis* (TV) infection in pregnant women on entry into prenatal care in an inner-city population; 2) compare conventional microscopic methods vs. culture techniques in diagnosing TV in both symptomatic and asymptomatic pregnant patients; and 3) correlate wet mount microscopic and microbiologic characteristics of varying manifestations of trichomoniasis.

*Methods:* One thousand two hundred sixty patients in an inner-city population were tested at entry into prenatal care for TV by saline wet mount and culture techniques. Other tests for lower genital tract infection were also performed. Vaginal symptoms were ascertained through standardized questioning prior to examination. Standard microscopic and microbiologic data were also obtained for analysis. Wet mounts were systematically examined and considered negative if no TV was identified in 10 high powerfields (HPFs). Cultures were inspected from days 4 to 7 or until positive results were obtained. Results were analyzed using McNemar's test for correlated proportions, chi-squared test, or Fisher exact test where appropriate.

*Results:* Culture and wet mount results were available in 1,175 patients. TV infection was documented by one or both techniques in 110/1,175 (9.4%). Culture methods detected 105/110 (94.5%) of all patients while wet mount detected 90/110 (73%) (*P* <0.001). Vaginal symptoms were present in only 20/110 patents (18.2%). Among asymptomatic patients, culture detected 94% while wet mount detected 70% (*P* < 0.001). Among symptomatic patients, wet mount and culture were both effective and diagnosed 85% and 95% of infections, respectively (*P* = not significant). Patients with TV were more likely to have increased vaginal fluid wlaite blood cells (WBCs) and more severe vaginal flora disruption than uninfected controls. Subgroup analysis revealed wet mount-positive/culture-positive patients were more likely to have vaginal flora disruption, as evidenced by decreased lactobacilli and elevated vaginal pH, than wet mount-negative/culture-positive subjects. Coexistent infection rates were similar regardless of wet mount status. Elevated vaginal fluid WBCs were more common among patients with symptoms.

*Conclusions:* 1) Screening pregnant women for TV based solely on symptomatology is ineffective in this population; 2) culture techniques detected more infections than conventional microscopic evaluation; and 3) significant increases in vaginal fluid WBCs and altered vaginal flora are found in both symptomatic and asymptomatic TV, suggesting that both infestations have the potential to adversely affect pregnancy outcome. Studies on the influence of TV on pregnancy outcomes are ongoing.


Infectious Diseases in Obstetrics and Gynecology 1:228–234 (1994)
© 1994 Wiley-Liss, Inc.

### *Trichomonas vaginalis:* Diagnosis and Clinical Characteristics in Pregnancy

**R. Phillip Heine, James A. McGregor, Elisa Patterson, Ruth Parker, Deborah Draper, Janice French, and Ward Jones**

*Department of Obstetrics and Gynecology, University of Colorado Health Sciences Center, Denver, CO*

### Abstract

*Objective:* The objectives of this study were to 1) determine the prevalance and characterize the symptomatology of *Trichomonas vaginalis* (TV) infection in pregnant women on entry into prenatal care in an inner-city population; 2) compare conventional microscopic methods vs. culture techniques in diagnosing TV in both symptomatic and asymptomatic pregnant patients; and 3) correlate wet mount microscopic and microbiologic characteristics of varying manifestations of trichomoniasis.

*Methods:* One thousand two hundred sixty patients in an inner-city population were tested at entry into prenatal care for TV by saline wet mount and culture techniques. Other tests for lower genital tract infection were also performed. Vaginal symptoms were ascertained through standardized questioning prior to examination. Standard microscopic and microbiologic data were also obtained for analysis. Wet mounts were systematically examined and considered negative if no TV was identified in 10 high powerfields (HPFs). Cultures were inspected from days 4 to 7 or until positive results were obtained. Results were analyzed using McNemar's test for correlated proportions, chi-squared test, or Fisher exact test where appropriate.

*Results:* Culture and wet mount results were available in 1,175 patients. TV infection was documented by one or both techniques in 110/1,175 (9.4%). Culture methods detected 105/110 (94.5%) of all patients while wet mount detected 90/110 (73%) ($P < 0.001$). Vaginal symptoms were present in only 20/110 patents (18.2%). Among asymptomatic patients, culture detected 94% while wet mount detected 70% ($P < 0.001$). Among symptomatic patients, wet mount and culture were both effective and diagnosed 85% and 95% of infections, respectively ($P$ = not significant). Patients with TV were more likely to have increased vaginal fluid white blood cells (WBCs) and more severe vaginal flora disruption than uninfected controls. Subgroup analysis revealed wet mount-positive/culture-positive patients were more likely to have vaginal flora disruption, as evidenced by decreased lactobacilli and elevated vaginal pH, than wet mount-negative/culture-positive subjects. Coexistent infection rates were similar regardless of wet mount status. Elevated vaginal fluid WBCs were more common among patients with symptoms.

*Conclusions:* 1) Screening pregnant women for TV based solely on symptomatology is ineffective in this population; 2) culture techniques detected more infections than conventional microscopic evaluation; and 3) significant increases in vaginal fluid WBCs and altered vaginal flora are found in both symptomatic and asymptomatic TV, suggesting that both infestations have the potential to adversely affect pregnancy outcome. Studies on the influence of TV on pregnancy outcomes are ongoing. © 1994 Wiley-Liss, Inc.

Key words

Trichomoniasis, vaginitis, pathogenesis, prematurity, pregnancy

Despite advances in perinatal and neonatal care, preterm birth (PTB) remains the major cause of neonatal morbidity and mortality.[1] The causes of PTB are multifactorial; however, considerable evidence shows that common reproductive tract infections and/or associated inflammation play roles in

Address correspondence/reprint requests to Dr. James A. McGregor, University of Colorado Health Sciences Center, 4200 East Ninth Avenue, Box B198, Denver, CO 80262.

Received December 20, 1993

Accepted March 15, 1994

preterm labor (PTL), PTB, and premature rupture of membranes (PROM) in significant numbers of women.[2–4] Understanding possible causal pathogenic factors such as reproductive tract infection can allow for development of etiologic-based interventions to prevent at least some instances of PTB.

Case-controlled and cohort studies have linked vaginal infections associated with *Trichomonas vaginalis* (TV) to decreased gestational age at delivery and PROM.[5,6] Several smaller studies did not show these associations.[7,8] Recent results of a large multicenter (Vaginal Infection in Prematurity [VIP]) study, which included multivariate analysis to correct for potential confounding variables, confirm significant associations between TV infection and PROM, PTB, and low birth weight in diverse populations of women in the United States.[9] Further research is warranted to delineate and characterize possible causal roles of TV infection in the pathogenesis of prematurity. Recognition, treatment, and understanding of TV pathobiology during pregnancy can be important steps in the incremental reduction of PTB and PROM.

The purposes of this study were to 1) determine the prevalence and characterize symptomatology of TV in women in an inner-city population on entry into prenatal care; 2) compare culture to standard clinic-based microscopic wet mount techniques for the diagnosis of TV in symptomatic and asymptomatic women; and 3) correlate microbiologic and microscopic characteristics of TV in symptomatic and asymptomatic pregnant women.

### MATERIALS AND METHODS

### Patients

One thousand two hundred sixty women entering prenatal care at Denver General Hospital (Denver, CO) between July 1990 and September 1991 were enrolled in an integrated program for prematurity prevention. Prior to pelvic examination, lower reproductive tract symptoms were ascertained by standardized questions regarding 1) abnormal quantity or character of discharge, 2) vaginal or vulvar burning or irritation, and 3) urinary complaints. To correlate presentation and findings of TV with an uninfected control group, we analyzed as a control the next enrolled patient with no evidence of cervical or vaginal infection with TV, *Chlamydia trachomatis*, bacterial vaginosis (BV), *Neisseria gonorrhoeae*, or yeast species. This study was approved by the hospital's Investigational Review Board.

### Microbiology

At initial pelvic examination, specimens were obtained for various microbiologic, microscopic, and enzymatic assessments. A cotton-tipped swab was placed on the posterior fornix and cultured for TV (InPouch™ TV, Biomed Diagnostics, Santa Clara, CA). We previously demonstrated the InPouch™ TV to be comparable to the use of Diamond's media culture for identification of TV.[10] Another cotton-tipped swab from the upper lateral vaginal wall was tested for vaginal pH (ColorpHast Indicator Strips, pH 4–7, EM Science, Cherry Hills, NJ) and rolled onto a glass slide for Gram's stain evaluation for BV.[11] This swab was then placed into 50 μl of normal saline at room temperature for microscopic and amine test examinations. An endocervical sample was collected with a cotton-tipped swab and inoculated onto Thayer Martin agar (Remel, Denver, CO) for recovery of *N. gonorrhoeae*. A separate endocervical sample was tested for *C. trachomatis* by enzyme immunoassay (Pathfinder Chlamydia EIA Swab Collection System, Kallestad, Chaska, MN) using standard criteria. Further vaginal fluid specimens were collected from the posterior vaginal fornix and frozen at −70°C for measurement of vaginal fluid enzymes. Results of these tests will be reported elsewhere.

Cultures of *N. gonorrhoeae* and TV were held in a candle jar at room temperature and transported to the lab within 4 h of collection. Cultures for TV were examined from days 4 to 7 for characteristic protozoan motility and morphologic characteristics. Cultures for *N. gonorrhoeae* were incubated at 35°C with 5% carbon dioxide and the organism was identified by standard microbiologic techniques.[12]

### Microscopy

The vaginal sample in normal saline was examined immediately by wet mount slide for evidence of motile trichomonads, yeast pseudohyphae or bud forms, clue cells, lactobacillus morphotypes, and white blood cells (WBCs). At least 10 high power fields (HPFs) were examined by experienced clinicians. A drop of fluid was placed in 10% KOH and evaluated for release of amine odor, i.e., whiff test. The fluid was again examined for yeast forms.

**TABLE 1. Symptoms associated with TV infection**

| Symptom | N | Symptomatic patients (%) | All patients (%) |
|---|---|---|---|
| Discharge | 8 | 40 | 7 |
| Pruritus | 8 | 40 | 7 |
| Odor | 6 | 30 | 5 |
| Dysuria | 1 | 5 | 1 |
| Burning | 3 | 15 | 3 |

A wet mount was considered positive for TV when motile trichomonads were visualized. BV was diagnosed when 2 of the following 3 occurred: ≥20% of epithelial cells were clue cells, vaginal fluid pH ≥ 4.7, and/or a whiff test was positive.[13] In presence of TV, the diagnosis of BV was made only if ≥20% clue cells were present. As we previously obtained good correlation between clinic-based and Gram's stain diagnosis of BV, only a clinic-based diagnosis of BV was evaluated in this study.[14] WBCs were considered elevated if >5 were visualized on wet mount in each HPF examined; less than 1 lactobacillus morphotype per HPF was considered decreased.

### Statistical Analysis

McNemar's test for correlated proportion was used to compare the wet mount with culture for diagnosis of TV. Chi-squared or Fisher exact test was utilized to compare various microscopic and microbiologic findings between groups.

### RESULTS

Culture and wet mount results were available in 1,175 patients. TV infection was documented in 9.4% (110/1,175). BV and yeast were present in 32.9% and 17.8%, respectively. *C. trachomatis* was isolated in 7.9% of patients; culture of *N. gonorrhoeae* was positive in only 11 women (0.9%). Demographic information in regard to age, parity, and racial distribution of those infected with TV was similar to the entire population with the exception of a 50% increase in prevalence among the African-American population (19% vs. 30%). The mean age of subjects was 25 years; two thirds of the patients were multiparous.

Symptoms elicited at study entry are listed in Table 1. Only 18.2% (20/110) of TV-infected women presented with complaints. This was not significantly different from the 11% with symptoms in the uninfected control group. Discharge and pruritus were the most common symptoms; however, they occurred in fewer than 1 in 10 women with TV. When comparing gestational age at examination, symptomatic patients were significantly more likely to present in the 1st and 2nd trimesters than in the 3rd trimester (Table 2). In fact, only 4% (1/30) with documented 3rd trimester infection presented with vaginal complaints, while 33% and 24% presented with symptoms in the 1st and 2nd trimesters, respectively.

**TABLE 2. Proportion of women presenting with symptomatic TV in each pregnancy trimester**

| Weeks gestation | N | TV symptomatic patients (%) | TV wet mount-positive (%) |
|---|---|---|---|
| 0–14 | 19 | 33* | 71 |
| 14–28 | 51 | 24* | 78 |
| 28+ | 30 | 4 | 84 |

*$P < 0.05$ compared with symptomatic patients at 28+ weeks gestation. Wet mount comparisons in the different trimesters were not significantly different.

**TABLE 3. Comparison of wet mount vs. culture in the diagnosis of TV both with and without symptoms[a]**

| | Diagnosis (% positive) | | |
|---|---|---|---|
| | All (N = 110) | TV symptomatic (N = 20) | TV asymptomatic (N = 90) |
| Wet mount | 73* | 85 | 70* |
| Culture | 95 | 95 | 94 |

[a]Wet mount was inferior in asymptomatic patients but equivalent in symptomatic patients.
*$P < 0.001$ compared with culture diagnosis.

Comparison of diagnostic methods revealed that culture was superior to wet mount in all subjects diagnosed with TV (Table 3). Analysis of subgroups based on symptomatology demonstrated that culture and wet mount were not different in symptomatic patients (85% vs. 95%), while culture was superior (94% vs. 70%) in asymptomatic patients. Diagnostic methods were equivalent in all 3 trimesters (Table 2).

The microscopic and microbiologic findings characterizing TV infection are presented in Table 4. Symptomatic women were more likely to have

**TABLE 4. Results (%) of vaginal microscopic and microbiologic findings in varying manifestations of TV and an uninfected control group[a]**

| | TV (N = 110) | TV symptomatic (N = 20) | TV asymptomatic (N = 90) | Wet mount ⊕ culture ⊕ (N = 74) | Wet mount ⊖ culture ⊕ (N = 30) | No infection (N = 110) |
|---|---|---|---|---|---|---|
| WBC > 5/HPF[b] | 50** | 76** | 44*,** | 54** | 43* | 26 |
| Lactcobacilli <1/HPF | 75** | 71** | 75** | 81** | 60*,** | 32 |
| Vaginal pH >4.5 | 79** | 75** | 71** | 79** | 43*,** | 17 |
| Whiff test | 39** | 52** | 36** | 40** | 27** | 3 |
| Coexistent infection | | | | | | |
| Any | 63 | 57 | 63 | 60 | 57 | — |
| BV | 43 | 52 | 40 | 48 | 30 | — |
| Yeast | 24 | 10 | 28 | 21 | 27 | — |
| *Chlamydia* | 9 | 0 | 10 | 10 | 3 | — |

[a]Symptomatic patients were more likely to have elevated vaginal WBCs compared with asymptomatic patients. Wet mount-positive patients were more likely to have decreased lactobacilli and an elevated vaginal pH compared with wet mount-negative patients. Patients with any TV infection were more likely to have elevated vaginal fluid WBCs and altered vaginal flora compared with uninfected controls.
[b]High power field (×400).
*$P < 0.05$ comparison within groups (asymptomatic vs. symptomatic, wet mount ⊕ vs. wet mount ⊖). Other comparisons between groups were not significantly different.
**$P < 0.05$ comparison to no infection group. All comparisons reached significance.

increased vaginal WBCs compared with asymptomatic patients. Vaginal flora disruption, as evaluated by decreased lactobacilli, altered vaginal pH, or positive amine test, was not different between symptomatic and asymptomatic women. Although the prevalence of coexistent infections was not significantly different, a trend toward increased *C. trachomatis* or yeast infections was observed in the asymptomatic group ($P = 0.10$). Both symptomatic and asymptomatic patients were more likely to exhibit increased density of vaginal WBCs and disrupted vaginal flora compared with uninfected control women.

Wet mount-positive/culture-positive patients had a similar prevalence of increased vaginal WBCs compared with wet mount-negative/culture-positive patients. However, wet mount-positive women were more likely to have disruption of the vaginal flora, as evidenced by a decrease in lactobacilli and an increase in vaginal pH. Coexistent infection rates were similar, but there was a trend toward an increase in BV in wet mount-positive vs. wet mount-negative women (48% vs. 30%, $P = 0.15$). Both wet mount-positive and wet mount-negative patients were more likely to have increased vaginal WBCs and vaginal flora disruption compared with uninfected controls.

The contributions of TV vs. effects from coexistent infections on vaginal pathology are shown in Table 5. Coexistent TV infection with BV created the most severe disruption of vaginal flora. TV alone exhibited significant microscopic microflora disruption compared with controls; however, TV alone or in combination with yeast rarely produced a positive whiff test. Furthermore, infection with yeast species did not significantly alter WBCs or lactobacillus outcomes; however, the sample size was small.

### DISCUSSION

In this study, 9.4% of inner-city Denver, CO, pregnant women presenting for prenatal care were infected with TV. Only 18.2% of TV-infected women complained of vaginitis-related symptoms prior to pelvic examination. For diagnosis, culture was superior to wet mount in this mostly asymptomatic population. Importantly, when compared with uninfected women, the occurrence of infection with TV either alone or in combination with other studied infections was associated with increased numbers of vaginal fluid WBCs and altered vaginal flora. Furthermore, symptomatic TV patients were more likely than asymptomatic patients to have increased vaginal fluid WBCs, while wet mount-

**TABLE 5. Vaginal microscopic and microbiologic findings for women with TV alone, TV with coexistent infections, and an uninfected control group[a]**

| | TV alone (N = 33) | Coexistent infections: Any (N = 65) | Coexistent infections: BV (N = 38) | Coexistent infections: Yeast (N = 14) | No infection (N = 110) |
|---|---|---|---|---|---|
| WBC > 5/HPFs | 55** | 53** | 68** | 43 | 26 |
| Lactobacilli <1/HPF | 55** | 83*,** | 97*,** | 50 | 32 |
| Vaginal pH >4.5 | 45** | 80*,** | 97*,** | 36 | 17 |
| Whiff test | 3 | 51* | 79*,** | 0 | 3 |

[a]Patients with BV or any infection were significantly more likely to have decreased lactobacilli, increased vaginal pH, and a positive whiff test compared with those with TV infection alone. Patients with TV alone or coinfected with BV were more likely to have elevated vaginal fluid WBCs and altered vagina flora compared with controls. TV alone did not exhibit a positive whiff test. TV combined with yeast was not different from controls.
*$P < 0.05$ compared with TV infection alone. Comparisons not noted were not significantly different.
**$P < 0.05$ compared with the no infection group. Comparisons not noted were not significantly different.

positive/culture-positive patients had more vaginal flora disruption than their wet mount-negative/culture-negative counterparts. Microscopic analysis also revealed that coexistent infection of TV with BV was associated with the greatest disruption of vaginal flora.

The TV prevalence rate in this study is consistent with the 12.6% rate obtained in the large multicenter VIP study.[9] Similar populations have documented TV infestation rates as low as 3% and as high as 48%.[15,16] Our findings appear to be representative of infection rates in many urban populations. The increased prevalence among African-American women is consistent with previous reports.[17] Other prior reported associations such as infection in older women[18] and/or coexistent infection with *N. gonorrhoeae* were not noted.[19] However, we had an extremely low prevalence of *N. gonorrhoeae*. Importantly, TV was more commonly identified than either *N. gonorrhoeeae* or *C. trachomatis*, for which we routinely screen in our obstetric population.

Two prior reports describe symptoms in 62–91% of TV-infected pregnant women.[20,21] Neither of these studies rigorously or systematically assessed symptoms prior to diagnosis. One previous study involving pregnant women evaluated symptoms by severity; fewer than 20% of patients with TV had "marked" symptoms, the majority being classified as "slight."[22] In each of these studies, symptomatology was elicited after identification of TV. Our documentation of symptoms prior to vaginal examination and the commonly held belief that some vaginal symptomatology is normal in pregnancy could explain such divergent results. This latter suggestion is supported by our findings of increased symptomatic infections in the 1st and 2nd trimesters, although symptoms in the 1st trimester were present in only 33% of women. Overall, in our population, TV-ascribable symptoms were uncommon and could not be used to identify women for selective examination.

Reported sensitivities of wet mount in the diagnosis of TV vary from 35% to 82%.[23,24] Our overall sensitivity approaches the optimal rate; smears were reviewed by practiced clinicians who visualized 10 or more HPFs prior to reaching a negative or positive diagnosis. In clinical practice, numerous examiners with varying levels of expertise utilizing nonstandardized techniques will likely identify fewer instances of TV infection. Even under our optimized research conditions, culture was superior to wet mount in women presenting with asymptomatic TV infection.

The classic microscopic finding of elevated numbers of vaginal fluid WBCs associated with TV was confirmed in this study. The degree of abnormality, however, was different from prior work. Previous work revealed increased vaginal WBCs in approximately 75% of patients infected with TV,[25] while only half of our patients exhibited elevations. A ratio of WBCs to epithelial cells of > 1 was previously used to define elevation, while the present study used absolute numbers. Such differences are probably not clinically relevant, but for research purposes, absolute numbers are undoubtedly more reproducible. We suggest that future studies use cell counting chambers to more accurately measure inflammatory cells in vaginal fluid.

In this study, both symptomatic and asymptomatic TV was associated with increased vaginal fluid WBC numbers. Subgroup analysis revealed that symptomatic patients were more likely than their asymptomatic counterparts to have elevated vaginal fluid WBCs. This suggests that immunologic activation and inflammation are important for development of symptoms. Strain-variable antigenicity is a potential explanation; however, experiments evaluating virulent vs. avirulent strains have shown no demonstrable differences to date.[26] More likely explanations are the increased production of neutrophil chemoattractant factors exhibited by virulent TV strains in vitro[27] and increased concentrations of microorganisms. Whatever the etiology, host response likely participates in possible TV-related adverse pregnancy outcomes.

The majority of previous investigations concur with the association of TV and altered vaginal flora.[15,16] TV infection alone was associated with decreased levels of lactobacilli; however, coinfection with BV appeared largely responsible for elevated vaginal pH and positive amine test associated with TV. Our study showed that fewer than 50% of patients with TV alone or TV with yeast had a vaginal pH of greater than 4.5, and only 1 of 47 had a positive amine test. Clearly, BV should be suspected in TV patients with altered vaginal flora. Prior teaching that these conditions are mutually exclusive appears incorrect. Future investigations regarding TV or BV associations with adverse pregnancy outcome should account for effects of combined infections.

Wet mount-positive/culture TV-positive patients demonstrated greater vaginal flora disruption than did wet mount-negative/culture-positive patients; however, the numbers of patients with increased vaginal fluid WBCs were similar. Previous work suggests that decreased parasitic burden is responsible for wet mount negativity.[19] Therefore, our findings suggest that vaginal floral disruption is associated with trichomonad numbers, while host responsiveness as manifested by increased numbers of vaginal fluid WBCs is less directly inoculum-related.

These findings have relevance to studies evaluating untoward effects of TV infection during pregnancy. Research studies linking TV with PTB and PROM used microbiologic methods to identify infection; in contrast, most clinicians caring for pregnant women use symptoms or grossly abnormal vaginal discharge to identify TV using wet mount microscopy. Obviously, the use of microbiologic-based methods can lead to identification of more women at risk for TV-associated pregnancy morbidity; future work will show whether symptomatic or wet mount-positive women are at greater risk than presumably more lightly infected wet mount-negative women. We speculate that 1) densities of infecting microorganisms, 2) individual infesting strains' abilities to produce virulence factors, and 3) nature of host responses may all contribute to the pathogenesis of TV-related adverse pregnancy outcome. In this study, both wet mount-positive and wet mount-negative TV-infected women demonstrated increased numbers of vaginal fluid WBCs. This suggests that even low density infections are associated with possibly damaging local host perturbations, which could increase risks of pregnancy morbidity. If host response is the most important determinant of TV-associated morbidity, then universal screening and treatment may be most appropriate.

In summary, TV was common among pregnant women receiving publicly supported antenatal care in Denver, CO. Under optimized research conditions, wet mount diagnosis was inferior to culture for identification of TV except in the minority of patients who were symptomatic. Because symptoms were so infrequent, a program involving screening of symptomatic patients will be ineffective. The optimal testing method among culture or wet mount requires correlation of varying manifestations with pregnancy outcome. TV alone, or in combination with other infections, regardless of symptomatology or wet mount positivity, created considerable disruption of the vaginal flora and elevated vaginal WBCs. This suggests that all manifestations of TV have the potential to create complications in pregnancy, and screening methods that identify the majority of cases appear justified.

### REFERENCES

1. Lee K, Paneth N, Gartner LM, Pearlman MA, Gruss L: Neonatal mortality: An analysis of the recent improvement in the United States. Am J Public Health 70:15–21, 1980.
2. McGregor JA: Prevention of preterm birth: New initiatives based on microbial-host interactions. Obstet Gynecol Surv 43:1–14, 1988.
3. Romero R, Mazor M, Wu YK, et al.: Infection in the pathogenesis of preterm labor. Semin Perinatol 12:262–279, 1988.
4. McGregor JA, French JI, Seo K: Adjunctive clindamycin therapy for preterm labor: Results of a double-blind, placebo-controlled trial. Am J Obstet Gynecol 165:867–875, 1991.
5. Hardy PH, Nell EE, Spence MR, et al.: Prevalence of six sexually transmitted disease agents among pregnant inner-city adolescents and pregnancy outcome. Lancet 2:333–337, 1984.
6. Minkoff H, Grunebaum AN, Schwarz RH, et al.: Risk factors for prematurity and premature rupture of membranes: A prospective study of the vaginal flora in pregnancy. Am J Obstet Gynecol 150:965–972, 1984.
7. Mason PR, Brown ML: Trichomoniasis in pregnancy. Lancet 1025–1026, 1990.
8. Ross SM, Van Middlekoop A: *Trichomonas* infection in pregnancy: Does it affect perinatal outcome? S Afr Med J 63:566–567, 1983.
9. Cotch MF: Vaginal Infections in Prematurity Study Group. Carriage of *Trichomonas vaginalis* is associated with adverse pregnancy outcome. Presented at the 30th Interscience Conference on Antimicrobial Agents and Chemotherapy, Abstract 681, Atlanta, Georgia, October 21–24, 1990.
10. Draper DL, Parker R, Patterson E, et al.: Detection of *T. vaginalis* in pregnant women with the InPouch® TV culture system. J Clin Microbiol 31(4):1016–1018, 1993.
11. Nugent RP, Krohn MA, Hillier SL: Reliability of diagnosing bacterial vaginosis is improved by a standardized method of Gram's stain interpretation. J Clin Microbiol 29:297–301, 1991.
12. Lennette EH, Balows A, Housler WJ Jr, Shadomy MG (eds): Manual of Clinical Microbiology. 4th Ed. American Society for Microbiologists, 1985.
13. Krohn MA, Hillier SL, Eschenbach DA: Comparison of methods for diagnosing bacterial vaginosis among pregnant women. J Clin Microbiol 27:1266–1271, 1989.
14. McGregor JA, French JI, Jones W, et al.: Association of cervicovaginal infections with increased vaginal fluid phospholipase $A_2$ activity. Am J Obstet Gynecol 167: 1558–1594, 1992.
15. deLouvou J, Hurley R, Stanley VC: Microbial flora of the lower genital tract during pregnancy. J Clin Pathol 28:731–735, 1975.
16. Kite EK, Hesseltine HC, Goldstein L: A study of the bacterial flora of the normal and pathologic vagina and uterus. Am J Obstet Gynecol 53:233–240, 1947.
17. Cotch MF, Pastorek JG, Nugent RP, et al.: Demographic and behavioral predictors of *Trichomonas vaginalis* infection among pregnant women. Obstet Gynecol 6:1067–1092, 1991.
18. Burch TA, Ries CW, Reardon LV: Epidemiologic studies of human trichomoniasis. Am J Trop Med Hyg 8:312–318, 1959.
19. Fouts AC, Kraus SJ: *Trichomonas vaginalis:* Reevaluation of its clinical presentation and laboratory diagnosis. J Infect Dis 141; 137–143, 1980.
20. Bramley M: Study of female babies entering confinement with vaginal trichomoniasis. Br J Vener Dis 52:58–62, 1976.
21. Robinson SC, Michandani G: Observation on vaginal trichomoniasis. Am J Obstet Gynecol 91:1005–1012, 1965.
22. Wisdom AR, Dunlop EMC: Trichomoniasis. Study of the disease and its treatment in women. Br J Vener Dis 41:90–96, 1965.
23. Laughlin M, Tidwell B, Finley R, Cleary J, Lushbaugh W: A double blind placebo-controlled troal of single-dose intravaginal vs. single-dose oral metronidazole in the treatment of *Trichomonas vaginalis.* Presented at the 32nd Interscience Conference on Antimicrobial Agents and Chemotherapy, Abstract 225, Anaheim, California, October 11–14, 1992.
24. McMillian A: Laboratory diagnostic methods and cryopreservation of trichomonads. In Honigberg BM (ed): Trichomonads Parasitic in Humans. New York: Spring Hill, pp 297–310, 1990.
25. Rein MF: Clinical manifestations of urogenital trichomoniasis in women. In Honigberg BM (ed): Trichomonads Parasitic in Humans. New York: Spring Hill, pp 225–234, 1990.
26. Su-Lin KE, Honigberg BM: Antigenic analysis of *Trichomonas vaginalis* strains by quantitative fluorescent antibody methods. Z Parasitenkd 69:162–181, 1983.
27. Mason PR, Forman L: Polymorphonuclear cell chemotaxis to secretions of pathogenic and nonpathogenic *Trichomonas vaginalis.* J Parasitol 68:457–462, 1982.
